# Differences between perceived age and chronological age in women: A multi‐ethnic and multi‐centre study

**DOI:** 10.1111/ics.12727

**Published:** 2021-08-08

**Authors:** Rainer Voegeli, Rotraut Schoop, Elodie Prestat‐Marquis, Anthony V. Rawlings, Todd K. Shackelford, Bernhard Fink

**Affiliations:** ^1^ DSM Nutritional Products Kaiseraugst Switzerland; ^2^ Newtone Technologies Lyon France; ^3^ AVR Consulting Ltd Northwich UK; ^4^ Department of Psychology Oakland University Rochester Michigan USA; ^5^ Biosocial Science Information Biedermannsdorf Austria; ^6^ Department of Evolutionary Anthropology University of Vienna Vienna Austria

**Keywords:** chronological age, cross‐cultural, ethnicity, face, perceived age, perception, women

## Abstract

**Objective:**

Accuracy in assessing age from facial cues is important in social perception given reports of strong negative correlations between perceived age and assessments of health and attractiveness. In a multi‐ethnic and multi‐centre study, we previously documented similar patterns of female facial age assessments across ethnicities, influenced by gender and ethnicity of assessors.

**Methods:**

Here we extend these findings by examining differences between estimated age from digital portraits and chronological age (Δ age) for 180 women from three age groups (20–34, 35–49, 50–66 years) and five ethnicities (36 images of each ethnicity, assessed for age on a continuous scale by 120 female and male raters of each ethnicity).

**Results:**

Across ethnicities, Δ age was smallest in French assessors and largest in South African assessors. Numerically, French women were judged oldest and Chinese women youngest relative to chronological age. In younger women, Δ age was larger than in middle‐aged and older women. This effect was particularly evident when considering the interaction of women's age with assessor gender and ethnicity, independently and together, on Δ age.

**Conclusion:**

Collectively, our findings suggest that accuracy in assessments of female age from digital portraits depends on the chronological age and ethnicity of the photographed women and the ethnicity and gender of the assessor. We discuss the findings concerning ethnic variation in skin pigmentation and visible signs of ageing and comment on implications for cosmetic science.

## Graphical abstract

We examined differences between perceived age from digital portraits and chronological age of women from three age groups and five ethnicities; the images were cross‐culturally assessed for age by female and male raters of the same five ethnicities. Accuracy of the assessment of a woman's chronological age from a digital portrait depends on the ethnicity of the photographed person and the ethnicity of the assessor. Younger women were judged less accurately than middle‐aged and older women, possibly because of the absence of visual signs of ageing.

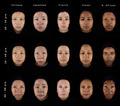

## Résumé

**Objectif:**

La capacité à évaluer l’âge d’un visage avec exactitude en fonction de ses caractéristiques est important dans sa perception sociale. En effet, des corrélations négatives fortes ont été rapportées entre l’âge perçu d’un visage d’une part, et sa santé et attractivité d’autre part. Dans le cadre d’une étude multi‐ethnique et multicentrique, nous avons déjà documenté, dans une démarche similaire, comment la perception de l’âge de visages féminins entre différentes populations, est influencée par le genre et l’origine des évaluateurs.

**Methodes:**

Ici nous approfondissons ces premiers résultats par l’étude des différences entre l’âge estimé sur portraits numériques de 180 femmes issues de 3 groupes d’âges (20‐34, 35‐49, 50‐66 ans) et de 5 populations d’origine différente (36 images de chaque population) et leur âge réel (Δ âge), et ce par 120 évaluatrices et évaluateurs de chaque population évaluant l’âge des visages en utilisant une échelle continue.

**Resultats:**

Au sein des différentes populations d’évaluateurs, le Δ âge le plus faible a été trouvé chez les évaluateurs français et le plus élevé chez les évaluateurs sud‐africains. Sur portraits numériques, les femmes françaises ont été perçues comme étant les plus âgées et les femmes chinoises les plus jeunes, par rapport à leur âge réel. Chez les femmes les plus jeunes, le Δ âge a été plus élevé que chez les femmes d’âge moyen et les plus âgées. Ceci a particulièrement été le cas lorsque l’on considère les interactions entre l’âge des femmes évaluées, et le genre et l’origine des évaluateurs, de façon indépendante ou liée, avec le Δ âge.

**Conclusion:**

Aux travers des différentes analyses, nos résultats suggèrent que l’exactitude avec laquelle l’âge des femmes est évalué sur images numériques de leur visage, dépend de l’âge réel et de l’origine de ces femmes photographiées, ainsi que de l’origine est du genre de l’évaluateur. Nous discutons ces résultats en regard des variations de pigmentation cutanée et de signes visibles de l’âge entre les différentes populations et commentons les implications possibles pour les sciences cosmétiques.

## INTRODUCTION

The accurate perception of a person's age plays a role in clinical and non‐clinical settings given the assumption that people who are perceived to be older than their chronological age are less healthy [1]. Perceived age predicts survival and correlates with molecular markers of ageing [2, 3]. In addition to relationships with molecular measures, perceived age correlates with cognitive functioning after controlling for chronological age and gender [4]. Age estimation of unfamiliar faces can be accurate [5, 6], although overestimating the age of younger faces is common and evidence suggests that the age of older individuals is more likely underestimated than the age of younger individuals [7, 8, 9]. Some studies report greater accuracy of female than male assessors, in particular when rating older faces [10]. Older‐appearing faces appear more distinctive and memorable than younger‐appearing faces [11, 12].

Perceived age is a robust biomarker of ageing that varies with indices of skin and hair ageing [13, 14], even after controlling for chronological age. Gunn et al. [15] reported that women judged younger than their chronological age have full lips, avoid sun exposure, and possess genetic factors that prevent the development of grey hair, skin wrinkles, and blemishes. Although hair greying and facial wrinkling are prominent features of ageing, anatomical studies document bony remodelling of the skull throughout life. This includes clockwise rotation of the midface relative to the cranial base [16], posterior displacement of the maxilla [17], lateral inferior shifting of the (lateral and inferior) orbital rim, development of a larger orbital aperture [18], and shrinking of the mandible [19, 20, 21]. Associated with these skeletal changes are a decrease in facial height and an increase in facial width and depth together with coarsening of bony prominences [22]. The face appears flattened, the nose and ears increase in size [23], and the lips become thinner [24, 25]. The bony scaffolding of the skull with time impacts the overlying soft tissue and retaining ligaments of the face. Together with diminished thickness and elasticity of the skin, loss of subcutaneous facial fat, and decreased skin adherence, gravity‐assisted sagging creates the typical appearance of ageing including prominent folds around the nasolabial region, periorbital region, and jowl [19].

Age‐related transformations of the cranium in middle and later adulthood are less notable than changes in the soft tissue of the face [20]. Thus, in addition to bony remodelling, facial ageing is influenced by the deterioration of soft tissue, and studies indicate that facial wrinkles have a larger impact on perceived age than does cranial shape [26, 27]. Changes in skin surface topography and skin pigmentation are clear signs of cutaneous ageing influenced by intrinsic (e.g., chronological ageing) and extrinsic (e.g., environmental) factors [28, 29, 30] that affect assessments of age, health, and attractiveness [10, 31].

An obvious ethnic skin difference relates to colouration, both within and between human populations, which varies with melanin and haemoglobin. There is variation in the size, distribution, and autophagic degradation of melanosomes (which contain eumelanin and phaeomelanin) across ethnicities, producing variation in skin colouration, photo‐protection, and ageing [30, 31, 32, 33]. For example, lightly pigmented individuals have an earlier onset and more pronounced skin wrinkling and sagging signs, whereas darkly pigmented individuals have more pigmentary problems. Ethnicities with more darkly pigmented skin retain younger skin properties compared with more lightly pigmented ethnicities [34]. Differences in epidermal thickness, the dermal–epidermal junction involution, and fibrillin together with collagen architecture are observed in Black African skin more than in Eurasian and East Asian skin [35]. However, age‐related dermal architecture degradation can occur in all skin types [36, 37, 38, 39] leading to laxity, rhytides, and discolouration (see [40] for a recent intra‐ethnic comparison). While differences in melanin concentration render darkly pigmented individuals more vulnerable to uneven skin colouration (e.g., dark/brown spots), their thicker dermis may make facial wrinkles less noticeable [41]. Differences in skin characteristics between ethnicities affect the relative importance that skin features play in the perception of age [40], including evidence for ethnic variation among early menopausal women [42]. More recently, two photoaging skin phenotypes have been identified (atrophic and hypertrophic skin) but their contribution to the perception of skin age in different ethnic groups is as yet unknown [38, 43, 44].

In the present study, we examine cross‐cultural variation in differences between the estimated age of women from digital portraits and their chronological age (Δ age). Research on perceived age relative to chronological age has been mostly conducted within a given society, or by recording age assessments of several ethnicities from members of one ethnicity. Here, we investigate whether Δ age in women of one ethnicity is shared by members of different ethnicities, in addition to effects of assessor gender. We employ a mixed‐model approach, using the raw scores of nearly 52 000 age judgements to describe Δ age in women as a function of ethnicity and age, in addition to assessor ethnicity and gender. Thus, we extend our previous research on assessments of female age [45] as part of a multi‐centre and multi‐ethnic study in which female and male individuals identifying with one of five ethnicities (Chinese, French, Indian, Japanese, and South African) judged facial images of women within and across ethnicities.

## MATERIALS AND METHODS

### Study sample

We secured digital portraits of women and ratings of these images in five locations – Guangzhou (China), Lyon (France), New Delhi (India), Tokyo (Japan), and Cape Town (South Africa) – using experimental equipment and protocols that were part of a larger project on the perception of female physical appearance [45]. The image recording occurred from April 2019 to September 2019, and the image rating occurred from October 2019 to February 2020. All participants provided written informed consent, including consent for publication of images included in this article. The Reading Independent Ethics Committee (RIEC), Woodley (U.K.), and the ACEAS Independent Ethics Committee, Ahmedabad (India), approved the study.

In total, 526 women (“participants”) were recruited through local agencies and imaged: Chinese (*n* = 106), French (*n* = 105), Indian (*n* = 100), Japanese (*n* = 100), and South African (*n* = 115). Each sample included participants from the ages of 20–69 years, equally distributed around the mean ages of 10‐year cohorts. According to the Fitzpatrick scale (Fitzpatrick, 1975), with type I = lightest pigmentation and VI =darkest pigmentation, participants corresponded to the following photo‐types: Chinese II–IV, French II–III, Indian IV–V, Japanese II–IV, and South African V–VI (this assessment was made by skin experts at the respective study centre).

Participants were interviewed before recruitment and those who met one or more of the following criteria were excluded from participation: (i) currently pregnant or lactating, (ii) suffering from visible facial pathologies or skin disease, (iii) receiving treatment for skin disease, (iv) involved in another clinical investigation or having participated in such within the past 2 months, (v) having facial tattoos or permanent make‐up, (vi) having topically applied hydroquinone‐containing product within the last 3 months, (vii) having a history of facial cosmetic surgery, laser treatment, or application of Botox or hyaluronic acid–based fillers.

### Facial imaging

On the day before imaging, no facial cosmetic or dermatological products (including foundation and/or colour products) were allowed but participants could use their regular facial cleanser or soap. On the morning of the day of imaging, participants were instructed to wash their faces with lukewarm water and dry them with a soft towel. After arrival at the study centre, a technician cleaned the participant's face with a cotton pad soaked with distilled water of ambient temperature and let it dry for 20 min. Facial adornment and glasses were removed for imaging. Before taking photographs, participants were acclimatized for 30 min at 21 ± 1°C and 45 ± 10% relative humidity.

**FIGURE 1:**
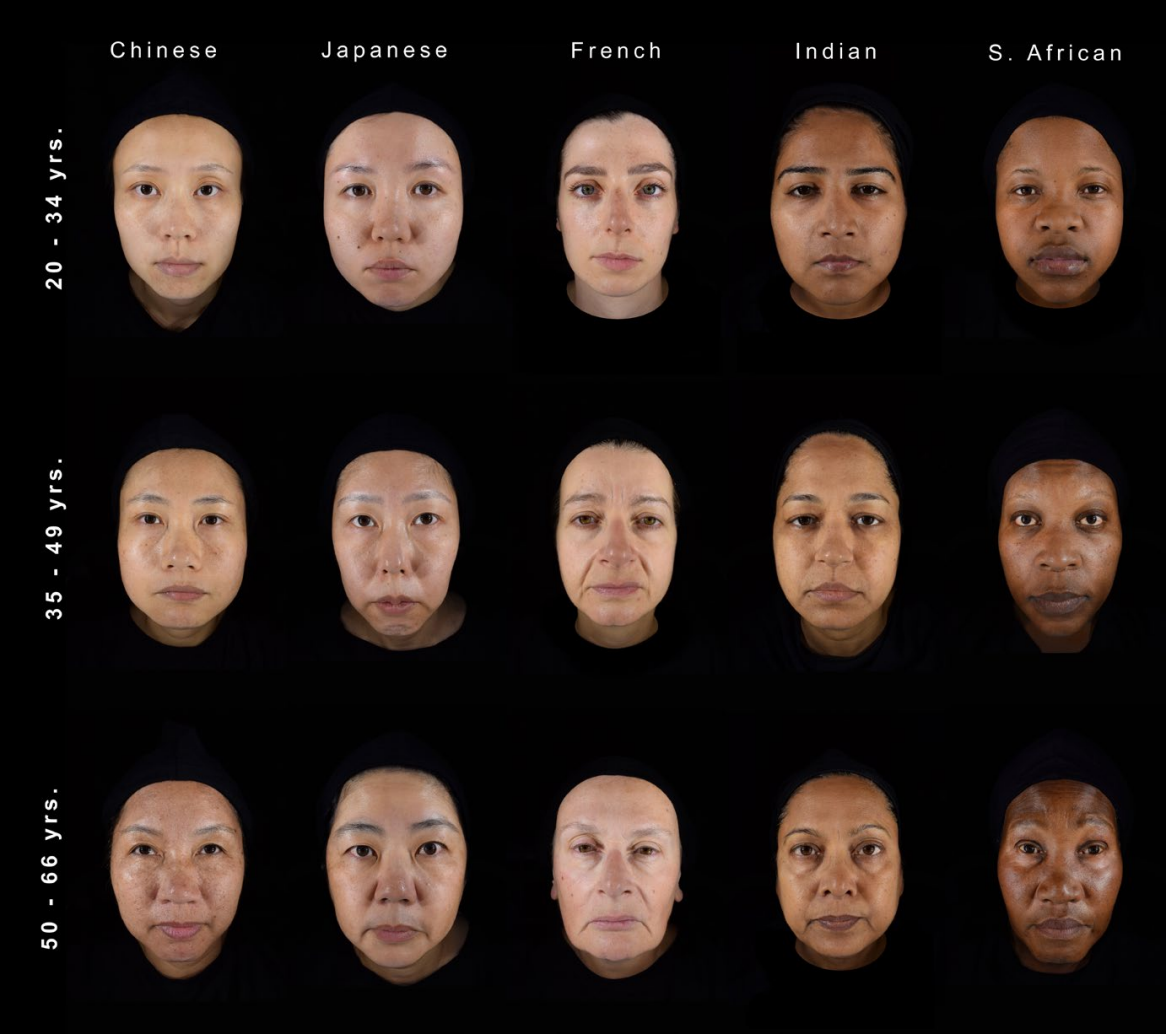
Sample images of female participants from five study locations and three age groups, respectively

Participants wore identical black hairbands and black capes to cover features that might affect facial assessments (e.g., head hair, chest, or clothes) (Figure 1). Their faces were imaged in frontal view, with eyes open, and with a neutral facial expression using the ColorFace system (Newtone Technologies, Lyon, France). ColorFace captures high‐resolution (24 MPs, at a maximum image size of 6000 × 4000 pixels, JPEG file format) full‐face images without a chin rest using an in‐built single‐lens reflex (SLR) camera (Nikon D5300; Nikon Inc., Minato, Japan). Earplugs attached to the stand of the device ensured standardized positioning of participants’ faces, with a fixed distance between the lens and face. A horizontal reference line connecting the corners of the mouth was displayed on the facial image visualized in real time on a remote computer, which served as an additional control before image capture. ColorFace uses LED light sources on the left and right sides of the face. System settings were selected to reduce the flash intensity and increase the light sensitivity of the camera sensor to avoid disturbance of the participant during imaging. For the presentation of the rating study, earplugs were digitally removed from images, eyes were vertically aligned, and the visible area of the neck was standardized across images.

### Face ratings

A sample of 600 volunteers (299 females) (“assessors”) were recruited through local agencies in the same locations (and study centres) where the facial images were recorded. Assessors reported having lived in the respective location for at least two years. The assessors’ skin photo‐types (on the Fitzpatrick scale) were matched by skin experts of the study centres with that of imaged women in each of the five study locations. Thus, we had female and male assessors of five ethnicities (*n* = 120 per location) (Table 1). Each ethnic group included assessors from the ages of 20–66 years equally distributed around the mean ages of 15‐year cohorts. The differences in mean ages between adjacent groups were 15 ± 2 years (all *p*s < 0.001).

**TABLE 1:** Sociodemographic information and skin pigmentation of female and male assessors in the rating study

Ethnicity	Fitzpatrick skin phototype	Gender	*n*	Age ± SD [years]
Chinese	II–IV	Female	60	42.4 ± 12.6
Chinese	II–IV	Male	60	43.2 ± 12.7
French	II–III	Female	60	43.7 ± 13.5
French	II–III	Male	60	43.7 ± 12.9
Indian	IV–V	Female	60	43.1 ± 13.1
Indian	IV–V	Male	60	42.4 ± 13.1
Japanese	II–IV	Female	60	42.8 ± 12.7
Japanese	II–IV	Male	60	43.2 ± 13.2
South African	V–VI	Female	59	43.3 ± 13.7
South African	V–VI	Male	61	43.3 ± 13.7

Of the initial sample (*n* = 526), a subset of 180 images was selected for presentation in the rating study (Table 2), following a quality check for suitability of images for inclusion in the rating study. Three expert raters independently assessed the initial image set on a 4‐point scale (1 = *not acceptable*, 4 = *acceptable*) for problems with positioning (e.g., head tilted), visibility of neck, and artefacts due to digital removal of earplugs. Only images considered “acceptable” by all three raters were considered for subset selection (*n* = 382).

**TABLE 2:** Sociodemographic information and skin pigmentation of imaged female participants (*n* = 12 for each group, chronological age in years ± SD)

Ethnicity	Fitzpatrick skin phototype	Age group 20–34 years	Age group 35–49 years	Age group 50–66 years
Chinese	II–IV	27.9 ± 4.3	42.4 ± 4.5	57.5 ± 4.5
Japanese	II–IV	27.4 ± 5.0	42.5 ± 4.2	57.5 ± 5.2
French	II–III	27.0 ± 4.6	42.9 ± 3.9	57.8 ± 4.3
Indian	IV–V	27.7 ± 4.4	42.6 ± 4.1	57.7 ± 5.0
South African	V–VI	27.6 ± 4.0	42.5 ± 4.6	57.3 ± 5.1

Image selection was randomly stratified for participant/assessor ethnicity and assessor gender and cohort; thus, of the available set of images, 36 images per ethnicity were assigned to female and male assessors of three cohorts by considering all possible factor combinations. The images were presented on colour‐calibrated, light‐shielded, 27‐inch LCD monitors (ColorEdge CG277, Eizo, Hakusan, Japan) with faces approximating natural size. The distance of the assessor to the monitor during assessment was 50–60 cm. Room conditions during the assessment were 21 ± 1°C and 45 ± 10% relative humidity with artificial light only.

Naive female and male assessors viewed facial images and provided judgments of perceived age, health, and attractiveness in blocks [45]. In the present study, we focus on age assessments and describe the procedure for that attribute. Assessors judged the images for age in a monadic presentation design (one after the other). Each assessor judged 90 randomly selected facial images, balanced across age groups. Age assessments were made using web‐based software (PhotoScale; Newtone Technologies, Lyon, France). The continuous scales ranged from 0 to 100, with age assessment provided in years. The serial order of images was randomized. The time for assessment was limited to 3–5 s. (before the image disappeared) to ensure viewing time was comparable across participants. Statements on the screen and the attributes were created in English and then translated into Mandarin, French, Hindi, Japanese, and Xhosa by native speakers and verified by back translation.

### Statistical analysis

We calculated the difference between the assessors’ estimated age and participants’ chronological age (Δ age) using the raw scores of age assessments and the date‐of‐birth information provided by women on the day of image recording.

A series of general linear mixed models (GLMMs) was performed with Δ age as the dependent variable, and with assessor ethnicity and gender, and participant ethnicity and age group as fixed effects (including interactions). Participant and assessor were included as crossed, independent random effects (both *p*s < 0.001). The *p*‐values for the fixed and interaction effects were corrected for multiplicity using the Benjamini–Hochberg method for control of the false discovery rate [46]. The analysis was performed in *R* [47], using the packages *lme4* [48] and *lmerTest* [49].

## RESULTS

There were main effects of *assessor ethnicity* (but not gender), *participant ethnicity*, and *participant age group* (Table 3). Specifically, differences between estimated age and chronological age of all participants were largest for South African assessors (followed by Indian and Chinese, both comparisons *n*.*s*.) and smallest for French assessors (with South African > Japanese and French, both *p*s < 0.001). That is, independent of other factors, French assessors estimated participant age most accurately. In contrast, French women were judged oldest relative to their chronological age, followed by South African and Indian women (both comparisons *n*.*s*.) and Japanese and Chinese women (*p* < 0.01 and 0.001, respectively). Age group of the participants showed an effect on Δ age with larger differences for younger than for middle‐aged and older participants (both *p*s < 0.001; middle‐aged vs. older, *n*.*s*.).

**TABLE 3:** Main and interaction effects of assessor ethnicity and gender, and participant ethnicity and age group, on differences between perceived and chronological age (Δ age) of female portraits.

Factor	*F*	*DF* *	*p*
Assessor Gender (AG)	0.22	1, 583	0.64
Assessor Ethnicity (AE)	11.00	4, 583	<0.001
Participant Ethnicity (PE)	7.09	4, 165	<0.001
Participant Age Group (PA)	13.51	2, 165	<0.001
AG × AE	3.12	4, 583	<0.01
AG × PE	4.49	4, 51 643	<0.01
AG × PA	107.27	2, 51 644	<0.001
AE × PE	15.00	16, 51 638	<0.001
AE × PA	138.79	8, 51 643	<0.001
PE × PA	0.66	6, 165	0.73
AG × AE × PE	1.11	16, 51 634	0.34
AG × AE × PA	8.42	8, 51 635	<0.001
AG × PE × PA	0.39	8, 51 643	0.93
AE × PE × PA	5.45	32, 51 639	<0.001
AG × AE × PE × PA	0.96	32, 51 634	0.53

* Calculated using the Satterthwaite method.

**FIGURE 2:**
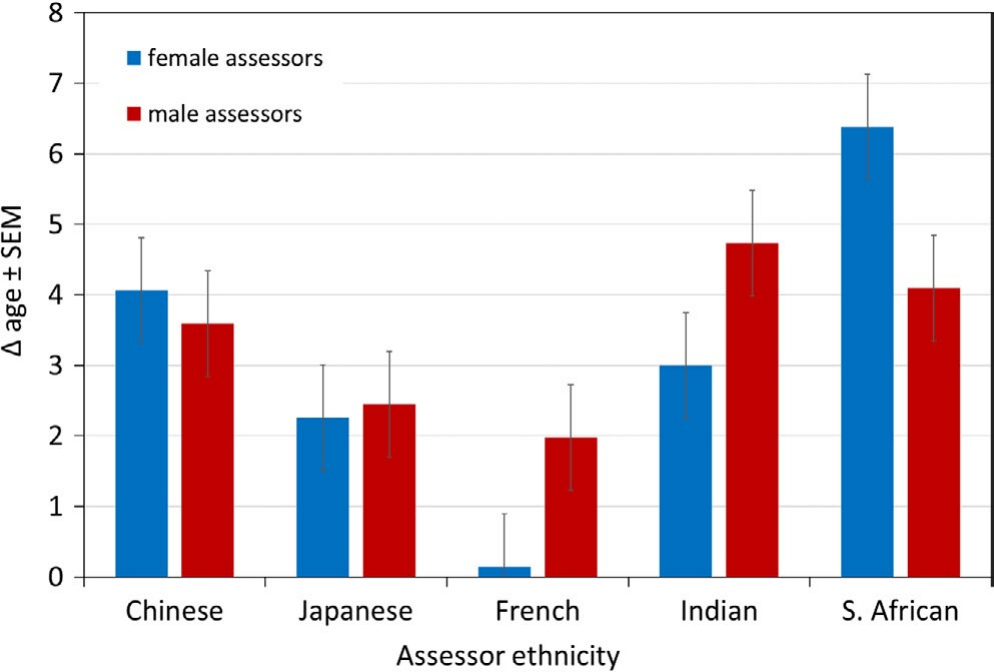
Difference between perceived age and chronological age (Δ age) of participants by assessor ethnicity and gender. Data are mean ± SEM

**FIGURE 3:**
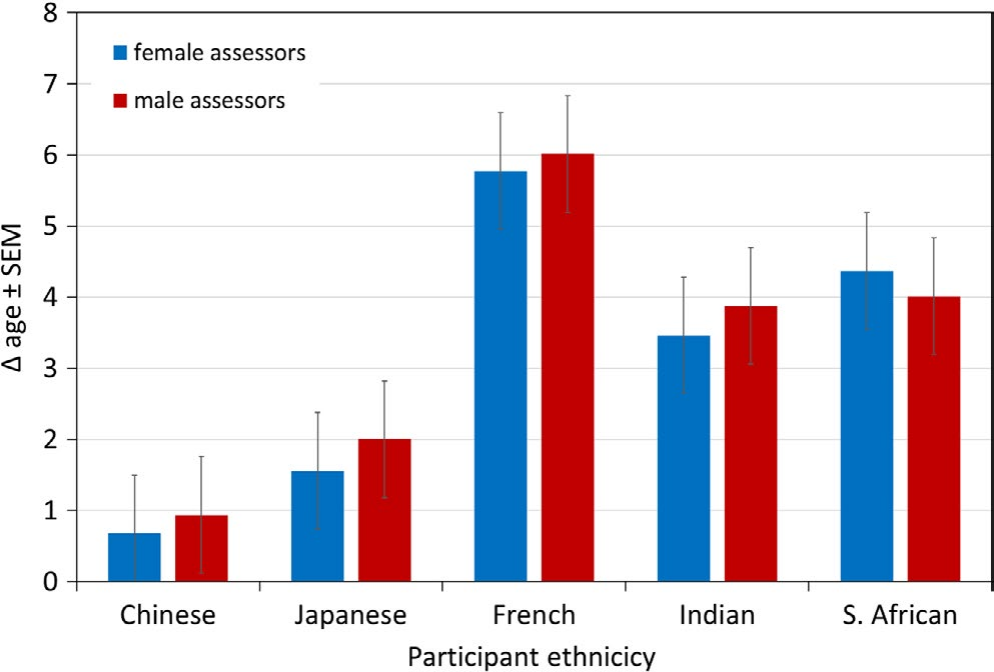
Difference between perceived age and chronological age (Δ age) of participant ethnicity by assessors. Data are mean ± SEM

We detected two‐way interactions on Δ age for *assessor gender and assessor ethnicity* (females: South African participants largest, French smallest, *p* < 0.001; 5 of 10 significant pairwise comparisons; males: Indian participants largest, French smallest, *p* < 0.05; 1 of 10 significant pairwise comparisons) (Figure 2) and *assessor gender and participant ethnicity* (in both genders: French participants largest, Chinese smallest, *p* < 0.001; females 5 of 10 and males 4 of 10 significant pairwise comparisons) (Figure 3).

**FIGURE 4:**
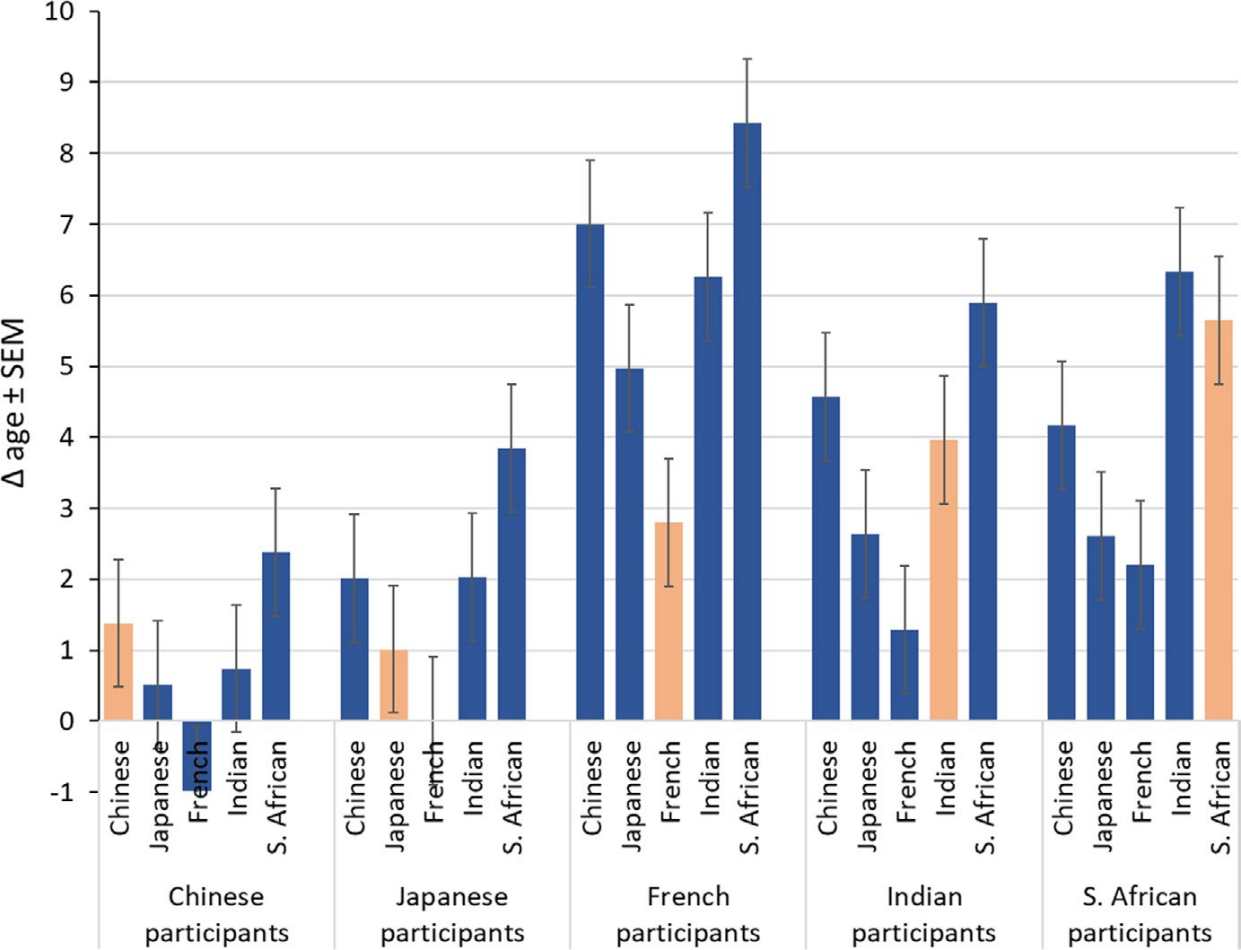
Difference between perceived age and chronological age (Δ age) of participant ethnicity by assessor ethnicity, orange bars indicate intra‐ethnic assessments. Data are mean ± SEM

In addition, there was an interaction of *assessor ethnicity and participant ethnicity* (Chinese, Japanese, and South African assessors: French > Indian > South African > Japanese > Chinese; French assessors: French > South African > Indian > Japanese > Chinese; Indian assessors: South African > French > Indian >Japanese > Chinese; 28 of 50 pairwise comparisons were different at *p* < 0.05 or lower) (Figure 4). Numerically, Δ age for intra‐ethnic assessments was largest for South African > Indian > French > Chinese > Japanese assessors.

**FIGURE 5:**
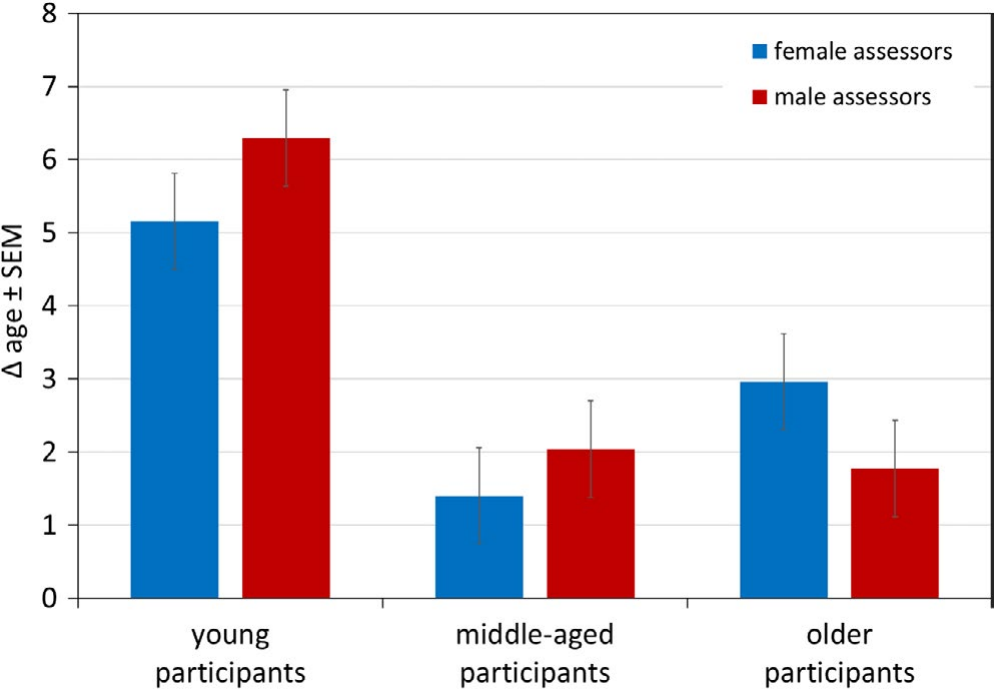
Difference between perceived age and chronological age (Δ age) of participant age groups by assessors. Data are mean ± SEM

There was an interaction of the *assessor gender and participant age group* (females: younger > older > middle‐aged, males: younger > middle‐aged > older; for both sexes pairwise comparisons including younger participants *p* < 0.05 or lower, but middle‐aged vs. older *n*.*s*.). For younger female participants Δ age males > females (*p* > 0.05), for middle‐aged women *n*.*s*., and for older participants females > males (*p* < 0.05) (Figure 5).

**FIGURE 6:**
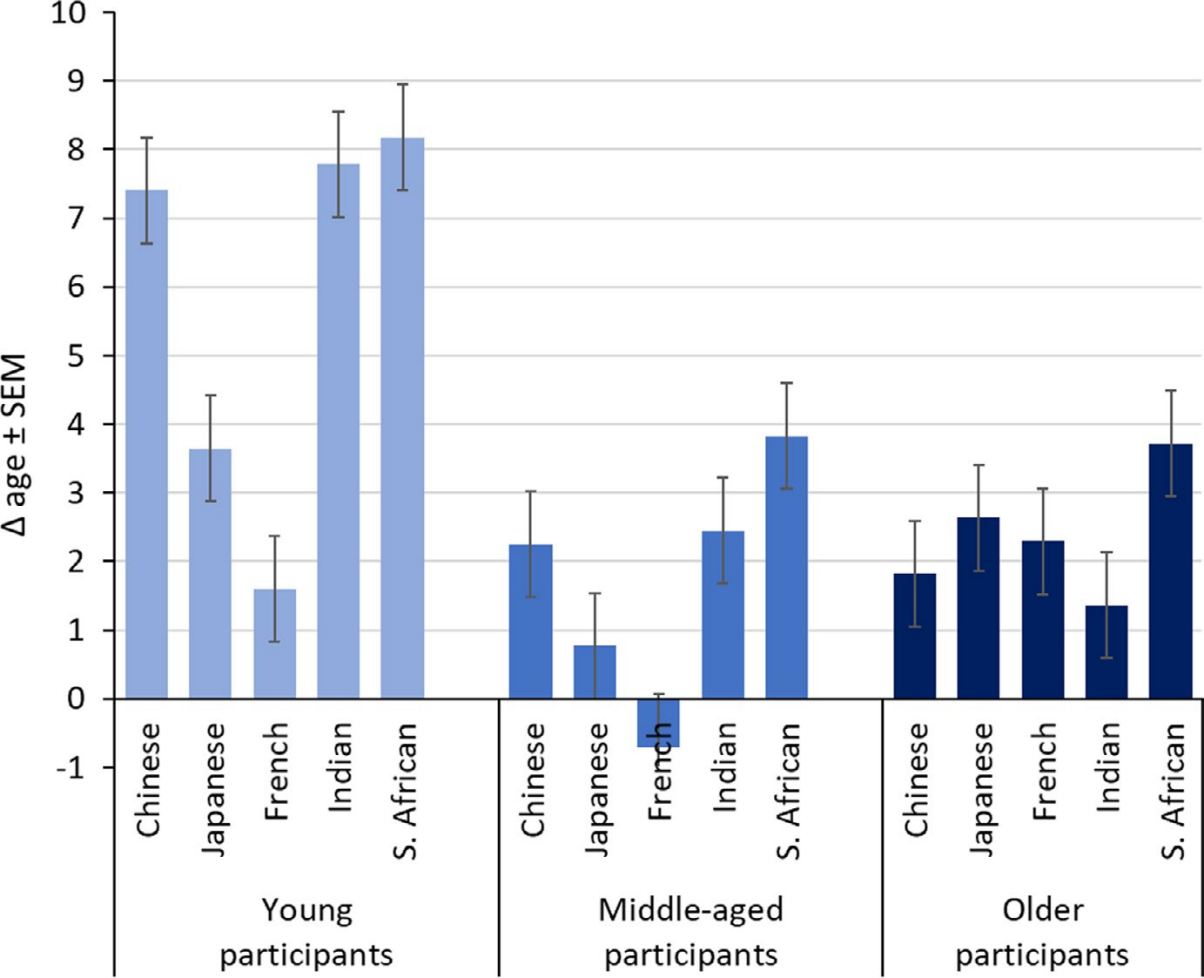
Difference between perceived age and chronological age (Δ age) of participant age groups by assessor ethnicity. Data are mean ± SEM

*Assessor ethnicity and age group* showed an interaction with Δ age across assessor ethnicity that was more pronounced for younger participants’ faces (a span between the means of up to ~7 years) than for middle‐aged participants (up to ~4 years) and older participants (up to ~2 years) (Figure 6). In younger participants, Δ age was largest for South African, Indian, and Chinese assessors (pairwise comparisons *n*.*s*.) with differences from Japanese and French assessors, respectively (*p*s < 0.001). The pattern was similar in middle‐aged participants, with differences especially for South African assessors > French and Japanese (both *p*s < 0.001) and South African, Indian and Japanese assessors vs. French at *p* < 0.001, respectively. In older participants, Δ age for South African > Chinese (*p* < 0.05) and Indian (*p* < 0.01) assessors. In total, 13 of 30 pairwise comparisons were significant at *p* < 0.05 for the interaction of the assessor ethnicity and participant age group. The interaction of the participant ethnicity and participant age group was not statistically significant.

**FIGURE 7:**
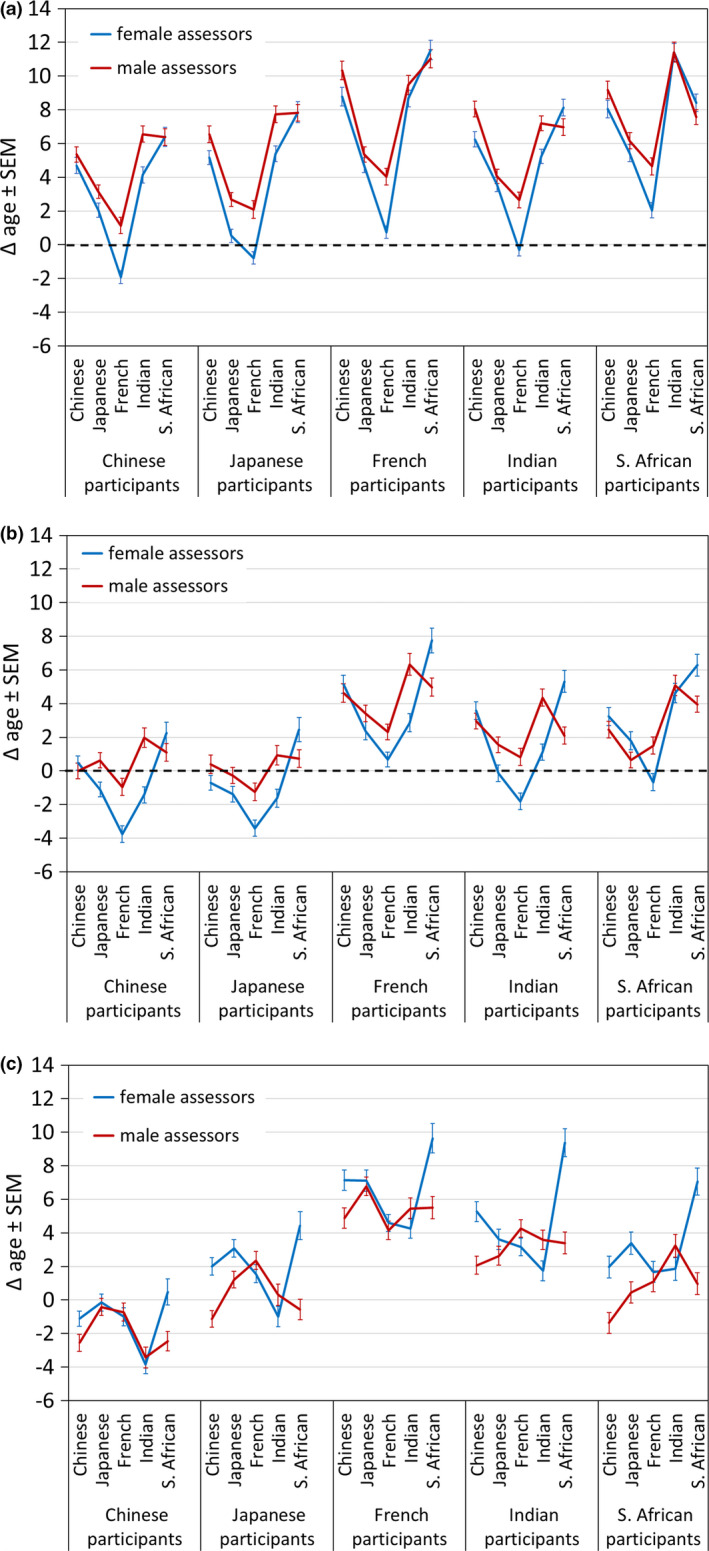
Difference between perceived age and chronological age (Δ age) by participant ethnicity, assessor ethnicity and gender for younger (a), middle‐aged (b), and older (c) participants. Data are mean ± SEM

Two of four three‐way interactions (both including assessor ethnicity and participant age group) were significant (Table 3). The interaction between assessor gender, assessor ethnicity, and participant age group suggests similar Δ ages of female and male assessors for participants across ethnicities, independent of participant age group as there were few age‐specific gender differences detected. In younger participants, Δ age was larger for female than for male assessors (*p* < 0.05). All other female–male comparisons were *n*.*s*. In middle‐aged participants, Δ age was larger for Indian male than Indian female assessors (*p* < 0.05), and, in older participants, Δ age for South African female assessors was larger than in South African male assessors. There were similar patterns across assessor ethnicities and assessor gender for younger participants, but different female–male patterns for targets of the middle‐aged and older women (Figure 7a–c).

The interaction of assessor ethnicity and participant ethnicity in this age group showed 4 of 50 significant pairwise comparisons for Δ age (*p* < 0.05 or lower). In middle‐aged participants, Δ age for male Indian assessors was largest (with differences from Japanese and French; *p*s < 0.05) whereas in female assessors Δ age was largest in South African assessors than for assessors from other ethnicities (*p*s < 0.05). The interaction of assessor ethnicity and subject ethnicity in this age group showed 6 of 50 significant pairwise comparisons for Δ age (*p* < 0.05 or lower). In the group of older participants, Δ age was highest for female South African assessors (with differences from all other assessors, *p*s < 0.05). Male assessors across ethnicities of older participants did not show a significant difference. The interaction of assessor ethnicity and subject ethnicity in this age group showed 16 of 50 significant pairwise comparisons for Δ age (*p* < 0.05 or lower).

The four‐way interaction between the assessor gender, assessor ethnicity, participant ethnicity and participant age group was not statistically significant.

## DISCUSSION

The present study suggests cross‐cultural variation in differences between perceived age (assessed from digital facial photographs) and chronological age of women depending on (i) the ethnicity and age of the person being assessed and (ii) the ethnicity and gender of the assessor. In addition to the main effects of these variables on Δ age (with gender being an exception), there were several (two‐ and three‐way) interaction effects among these variables, with the largest effect sizes (mean differences) detected for interactions that included age (group) of participants. This suggests that, in addition to effects of participant ethnicity and assessor ethnicity and gender, the age of the target women plays a significant role in the accuracy of age assessments (although no four‐way interaction was detected). In the following discussion, we focus on the two‐way interactions and the patterns that emerged in our findings in consideration of effect sizes and to reduce the level of complexity in the interpretation of the study findings.

French assessors were most accurate in the assessment of female age, and this was especially found for female assessors. One could argue that the estimation of age is a human capacity independent of ethnicity but cultural variation in the efficiency of facial age judgments has been reported [50]. Also, the assessors were recruited in larger cities in five different geographical locations and were familiar with the typical (age‐related) facial appearance of other ethnicities. Although cross‐cultural variation in facial familiarity may contribute to the observed effect, the French accuracy in assessing facial age may reflect a tradition in cosmetic science and the rise in popularity of cosmetic use since the beginning of the 20th century [51]. The assessors in the present study were laypeople without expertise in facial appearance. It will be interesting to investigate if the effect of expertise tracks down to lay assessors due to an increased level of awareness and exposure to beauty topics and cosmetic‐related content.

While French female and male assessors were most accurate in estimating the age of women of other ethnicities, French women were judged least precisely (i.e., as older than they were), and this effect was especially evident among younger women (20–34 years). We consider it likely that a combination of factors is responsible for this finding. From a psychological perspective, facial familiarity may lead to being particularly critical with faces of one's ethnicity [52]. However, this should lead to more accurate assessment (and thus a smaller Δ age) compared with assessments of other ethnicities, but this was not found. The smallest Δ age was detected for Chinese women. In addition to the face familiarity effect [53], the lifestyle and habits of women with lighter pigmentation may impact the larger difference between the estimated and chronological age of French women. Frequencies of alcohol consumption and smoking, for example, might be higher in French women than in other ethnic groups, and this may account, in part, for differential expressions of visual signs of ageing. We did not test for differences between participant ethnicities in lifestyle and habits because the relatively small sample of women (*n* = 180) may lead to false conclusions. However, the adverse cutaneous effects of smoking, for example, are well documented [54, 55]. Considering age estimates of the same ethnicities only, the pattern of findings was different, with larger Δ ages for South African and Indian participants than for French, Chinese, and Japanese participants (in that order). This ranking of age perception was different to that reported by Flament et al. [40] (South African > Indian > Chinese > Japanese > French). The reasons for the discrepancy in findings between the two studies needs to be investigated, preferably based on quantitative measures (in addition to grading [23, 40]) of facial shape/skin features. This may help to disentangle psychological (e.g., in‐group favouritism, [56]) from real differences in age‐related features between ethnic groups.

Perhaps the most obvious effect on Δ age across ethnicities of participants and assessors was that of participant age group, which also resulted in two three‐way interactions (together with assessor/participant ethnicity and assessor gender). Female and male assessors of all five ethnicities judged younger participants to be older than their chronological age, i.e., Δ age was largest for the group of 20–34‐year‐old women. Overestimating the age of younger faces (and underestimating the age of older faces) has been reported [7, 8, 9]. One of the reasons for this effect might be the absence of distinctive (charismatic) features in the face. Older‐appearing faces have been reported to be more memorable than younger‐appearing faces because of their distinctiveness [11, 12], and age‐related cutaneous changes contribute to facial distinctiveness. This suggests the absence of age‐related changes in facial appearance in younger individuals makes it more difficult to assess them accurately for age compared with older individuals (for a discussion of potential causes of the common observation of overestimating facial age, see [7]). In the present study, there were similar patterns across assessor ethnicities regarding Δ age of female targets among younger participants (although the means of Δ age differed across participant ethnicity). This pattern changed with increasing age of the participant, and differences between female and male assessors became evident in some ethnicities (as suggested by the three‐way interactions). For example, in older participants, Δ age was largest, and female–male differences were largest for South African assessors and South African targets. A similar (albeit less pronounced) pattern was observed for Indian participants. Regarding the ‘visibility’ of age‐related skin changes, one could speculate that due to variation in skin phototype certain age‐related features are less visible in darkly pigmented phototypes. Thus, these features may have a selective impact on female and male estimates of women's age, influenced by gender‐specific psychology and preference. Considering the findings of the present study together with previously reported attractiveness and health assessments [45], we cannot exclude the possibility that ethnocentrism and implicit attitudes play a role [57, 58, 59], at least in some of the detected patterns. The largest Δ ages were detected for Indian assessors estimating the age of French and South African women, South African assessing French, Indian, and South African women, and Chinese assessing French women.

Previous research documents variation in the age of onset, severity, and concerns with skin ageing across ethnicities [32, 34, 60, 61], and ethnic differences have partly been explained by structural and functional differences of skin types [62]. Thus, in addition to questions of noticeability, signs of ageing occur later in skin of colour [32] possibly because of the higher melanin content and the dispersal of melanosome in more darkly pigmented skin [63]. Although higher melanin content correlates negatively with the onset and severity of photoaging in darker skin, there is also a greater risk of pigmentation alteration [32, 34]. Structural differences in skin include the stratum corneum [64, 65, 66], which is more compact in darkly pigmented skin, differences in epidermal thickness, and rete ridge convolution and extracellular matrix arrangement [35, 36, 37, 38, 67, 68, 69]. Collectively these and other, less‐well understood, ethnic differences in skin may contribute to systematic variation in self‐perception and in age estimation from third‐party panellists. Lightly pigmented women report being more strongly affected by signs of ageing in all facial regions and this concern is supported by studies investigating systematic retouching (digital removal) of skin features such as fine lines/wrinkles and hyperpigmentation [70, 71]. Hyperpigmentation is of greater concern with skin of colour and may contribute to the development of facial neoplasms and other textural irregularities [34, 72]. This is not exclusive to persons with skin of colour as senile lentigines are known in Caucasian subjects also [73, 74, 75]. Alexis et al. reported that >30% of Black women were not concerned by the presence of moderate/severe facial signs of ageing until the ages of 60–79 years [60]. Caucasian women reported greater severity of facial ageing than other ethnic groups, and Asian and Hispanic women fell between Caucasians and Black women.

The findings of the present study corroborate the suggestion that in more darkly pigmented women, facial signs of ageing affect age assessments more in older women than in younger women given that the largest differences between perceived and chronological age were found in female assessors for South African women ages 50–66 years. Also, French women were judged oldest for their age (i.e., Δ age was largest), a finding that may correspond with reports of earlier onset of facial signs of ageing in Caucasian women [60]. Flament et al. reported cross‐cultural variation in the size of the correlation between perceived age and chronological age with wrinkles and ptosis/sagging being the predominant features that contribute to perceived age variation across ethnicities [40]. Clinical signs of facial wrinkles and sagging together accounted for 100% of perceived age in French women, whereas in South African women, for example, the percentage of variation accounted for was 61%. Thus, wrinkles/sagging are the predominant features that influence facial age perception across ethnicities. However, there is variation in the significance of other clinical signs of ageing across ethnicities (cheek skin pores and discolouration), albeit to a much lesser extent, which contributes to cross‐cultural differences in perceived age. The lack of pigmentary changes in the French participants is surprising as discussed previously. Flament et al. had a sample of experts grading facial signs in addition to age assessments from naive panellists, which were aggregated (per country) for subsequent analysis [40]. In the present study, we based our analyses on raw scores of nearly 52 000 age judgements to describe Δ age in women as a function of ethnicity and age (in addition to assessor ethnicity and gender), which may provide a more accurate assessment of Δ age given the linear mixed model analysis accounted for independent random effects from participants and assessors. Neither the present study nor the Flament et al. study considered relationships between objective (technical) measures of skin and subjective estimates of female age. This is an avenue for future research which would provide valuable information about the impact of visible differences in the skin across ethnicities on the perception of age (and other attributes).

The present study focuses on perceived female age relationships with age‐ and ethnicity‐related changes in facial skin. Previous research suggested that age‐related soft tissue changes are more notable than transformations of the cranium [20]. Although the current study did not quantify facial shape information and investigate the relative effects of facial shape and skin on perceived age, it is plausible that both features affect age perception. However, whether the relative contribution of facial shape and skin to perceived age is similar across ethnicities remains to be investigated, preferably using one set of stimuli and corresponding information from participants and assessments as differences in the protocols across (independent) studies can limit conclusions. Age‐related facial shape changes may vary between ethnicities, and this may lead to ethnicity‐dependent stereotypic face assessments [76]. Such differences may partly be responsible for the Δ age differences between ethnicities, more specifically, the finding that Δ age was smallest in Chinese and Japanese women, larger in Indian and South African women, and largest in French women. Skin tone unevenness alone cannot account for this, which may reflect a combination of both (i) structural and functional differences in skin between ethnic groups, leading to variation in the development and visibility of ageing skin and (ii) ethnicity‐specific changes in facial shape, including ptosis/sagging. We contend that this interpretation is speculative and requires quantitative assessment of facial shape (e.g., through geometric morphometric methodology [77]) and facial skin to disentangle effects and thus explain ethnic variation in the perception of facial age.

From a cosmetic formulation perspective, the importance of correcting senescent melanocytes involved in defining the skin ageing phenotype, i.e., not just pigmentary changes, needs more consideration in all ethnicities [78, 79]. Also, ultraviolet (UV)B is especially important for lightly pigmented skin whereas protection against UVA, visible light, and infrared A may be helpful for all skin phototypes [80]. These approaches will reduce the appearance of skin ageing and the perception of chronological age.

In conclusion, the present study indicates cross‐cultural differences in perceived age. The accuracy of the assessment of a woman's chronological age from a digital portrait depends on the ethnicity of the photographed person, the ethnicity of the assessor, and whether the assessor is female or male. Younger women were judged less accurately than middle‐aged and older women, possibly because of the absence of visual signs of ageing. The finding of French women being judged oldest and Chinese (and Japanese) women youngest relative to chronological age may be due to stereotypic facial attributions and remains to be clarified in future interdisciplinary (anthropological, dermatological, and psychological) approaches to ethnic diversity. Collectively, the present study highlights the role of ethnicity (in addition to age and gender) in the assessment of female facial age. The importance of considering ethnic diversity in face research will continue to increase in basic and applied studies of physical appearance given increasing mobility and exposure to members of other ethnic groups in a globalized world.

## CONFLICTS OF INTERESTS

RV and RS are employees of DSM, BF and AVR are consultants to DSM and EP is an employee of Newtone Technologies. TKS states no conflict of interest.

## Supporting information


**Table S1** Descriptive statistics (mean and 95% CI) of differences between perceived age and chronological age (Δ age), separately for assessor ethnicity and gender, and participant ethnicity and age groups.
