# Supplementary material for: Differences between perceived age and chronological age in women: A multi‐ethnic and multi‐centre study
Source: Int J Cosmet Sci. 2021 Aug 8;43(5):547–60. doi: 10.1111/ics.12727 (PMC9291153; doi:10.1111/ics.12727)
Supplement: Supplementary file 1 — Table S1 Descriptive statistics (mean and 95% CI) of differences between perceived age and chronological age (Δ age), separately for assessor ethnicity and gender, and participant ethnicity and age groups. [file ICS-43-547-s001.docx]

**Supplementary Information**

Table S1. Descriptive statistics (mean and 95% CI) of differences between perceived age and chronological age (Δ age), separately for assessor ethnicity and gender, and participant ethnicity and age groups.

1. Younger participants

|  |  | **Participant ethnicity** | | | | |
| --- | --- | --- | --- | --- | --- | --- |
| **Assessor**  **Ethnicity / Gender** | | Chinese | Japanese | French | Indian | South African |
| Chinese | F | 4.69 [2.20, 8.19] | 5.18 [2.27, 8.25] | 8.78 [6.17, 12.16] | 6.25 [3.38, 9.37] | 8.05 [5.07, 11.06] |
| Chinese | M | 5.35 [2.29, 8.33] | 6.55 [3.57, 9.61] | 10.33 [7.43, 13.46] | 8.04 [5.03, 11.07] | 9.17 [6.54, 12.58] |
| Japanese | F | 2.04 [-0.94, 5.05] | 0.52 [-2.22, 3.76] | 4.71 [1.64, 7.63] | 3.57 [0.36, 6.34] | 5.43 [2.38, 8.36] |
| Japanese | M | 3.14 [-0.27, 5.71] | 2.69 [-0.55, 5.43] | 5.35 [2.31, 8.29] | 4.05 [0.96, 6.95] | 6.16 [2.85, 8.84] |
| French | F | -1.92 [-4.49, 1.50] | -0.78 [-3.65, 2.33] | 0.74 [-1.99, 3.99] | -0.33 [-3.07, 2.91] | 2.04 [-0.61, 5.38] |
| French | M | 1.14 [-1.69, 4.31] | 2.09 [-1.17, 4.83] | 4.03 [0.99, 6.99] | 2.66 [-0.21, 5.78] | 4.65 [1.92, 7.91] |
| Indian | F | 4.14 [1.31, 7.31] | 5.39 [2.63, 8.62] | 8.65 [5.65, 11.65] | 5.23 [2.41, 8.41] | 11.41 [8.39, 14.39] |
| Indian | M | 6.56 [3.51, 9.51] | 7.73 [4.48, 10.48] | 9.47 [6.76, 12.76] | 7.20 [4.29, 10.29] | 11.41 [8.47, 14.46] |
| S. African | F | 6.40 [3.59, 9.61] | 7.85 [4.98, 10.99] | 11.55 [8.37, 14.38] | 8.13 [5.04, 11.05] | 8.41 [5.01, 11.02] |
| S. African | M | 6.38 [3.11, 9.10] | 7.82 [4.73, 10.71] | 11.03 [7.90, 13.88] | 6.96 [4.32, 10.29] | 7.56 [4.73, 10.71] |

1. Middle-aged participants

|  |  | **Participant ethnicity** | | | | |
| --- | --- | --- | --- | --- | --- | --- |
| **Assessor**  **Ethnicity / Gender** | | Chinese | Japanese | French | Indian | South African |
| Chinese | F | 0.47 [-2.26, 3.73] | -0.72 [-3.62, 2.37] | 5.15 [1.94, 7.94] | 3.58 [0.33, 6.32] | 3.24 [0.44, 6.43] |
| Chinese | M | 0.00 [-2.87, 3.17] | 0.39 [-2.85, 3.18] | 4.63 [1.72, 7.76] | 2.96 [0.04, 6.07] | 2.44 [-0.42, 5.62] |
| Japanese | F | -1.10 [-3.95, 2.04] | -1.38 [-4.10, 1.89] | 2.38 [-0.49, 5.49] | -0.15 [-2.88, 3.10] | 1.79 [-1.50, 4.48] |
| Japanese | M | 0.63 [-2.79, 3.20] | -0.27 [-3.32, 2.66] | 3.41 [0.44, 6.42] | 1.56 [-1.27, 4.71] | 0.65 [-2.37, 3.61] |
| French | F | -3.75 [-6.54, -0.55] | -3.41 [-6.54, -0.55] | 0.67 [-2.48, 3.50] | -1.81 [-5.25, 0.73] | -0.67 [-3.57, 2.42] |
| French | M | -0.95 [-3.95, 2.05] | -1.24 [-4.40, 1.60] | 2.31 [-0.68, 5.31] | 0.83 [-2.42, 3.58] | 1.51 [-1.18, 4.82] |
| Indian | F | -1.42 [-4.06, 1.94] | -1.61 [-4.58, 1.42] | 2.86 [0.00, 6.00] | 1.12 [-1.97, 4.03] | 4.62 [1.52, 7.52] |
| Indian | M | 1.98 [-1.32, 4.68] | 0.94 [-2.13, 3.87] | 6.33 [3.26, 9.25] | 4.36 [1.49, 7.49] | 5.07 [2.24, 8.24] |
| S. African | F | 2.26 [-0.49, 5.53] | 2.46 [-0.19, 5.83] | 7.76 [4.67, 10.69] | 5.31 [2.11, 8.13] | 6.29 [3.36, 9.37] |
| S. African | M | 1.09 [-2.15, 3.83] | 0.73 [-1.95, 4.03] | 4.98 [2.38, 8.36] | 2.10 [-0.61, 5.37] | 3.96 [1.12, 7.10] |

1. Older participants

|  |  | **Participant ethnicity** | | | | |
| --- | --- | --- | --- | --- | --- | --- |
| **Assessor**  **Ethnicity / Gender** | | Chinese | Japanese | French | Indian | South African |
| Chinese | F | -1.11 [-3.53, 2.46] | 2.00 [-1.32, 4.67] | 7.14 [4.58, 10.57] | 5.27 [1.46, 7.45] | 1.96 [-1.09, 4.90] |
| Chinese | M | -2.55 [-5.60, 0.44] | -1.13 [-4.04, 1.99] | 4.89 [2.16, 8.19] | 2.07 [-0.85, 5.19] | -1.36 [-3.61, 2.43] |
| Japanese | F | -0.15 [-3.16, 2.82] | 3.07 [-0.45, 5.53] | 7.11 [3.97, 9.95] | 3.62 [0.87, 6.85] | 3.39 [-0.6, 5.38] |
| Japanese | M | -0.42 [-3.73, 2.25] | 1.21 [-1.21, 4.77] | 6.77 [4.01, 10.00] | 2.64 [-0.18, 5.80] | 0.46 [-3.05, 2.94] |
| French | F | -1.00 [-3.65, 2.33] | 1.56 [-1.43, 4.56] | 4.59 [1.45, 7.44] | 3.17 [-0.35, 5.64] | 1.68 [-0.56, 5.43] |
| French | M | -0.72 [-3.52, 2.48] | 2.36 [-0.76, 5.23] | 4.15 [1.55, 7.54] | 4.26 [1.03, 7.03] | 1.11 [-0.76, 5.24] |
| Indian | F | -3.84 [-6.77, -0.77] | -0.98 [-3.93, 2.07] | 4.27 [1.34, 7.34] | 1.74 [-0.95, 5.05] | 1.85 [-1.02, 4.98] |
| Indian | M | -3.42 [-6.23, -0.24] | 0.31 [-2.29, 3.71] | 5.46 [2.58, 8.58] | 3.59 [0.54, 6.53] | 3.23 [0.38, 6.38] |
| S. African | F | 0.48 [-2.06, 3.96] | 4.43 [0.59, 6.61] | 9.63 [6.80, 12.81] | 9.38 [5.45, 11.47] | 7.05 [3.33, 9.35] |
| S. African | M | -2.46 [-5.72, 0.26] | -0.57 [-3.06, 2.92] | 5.5 [2.42, 8.40] | 3.39 [1.04, 7.02] | 0.97 [-1.64, 4.34] |
